# Comparative Analysis of Protein Quantification Methods for the Rapid Determination of Protein Loading in Liposomal Formulations

**DOI:** 10.3390/pharmaceutics11010039

**Published:** 2019-01-18

**Authors:** Maryam T. Hussain, Neil Forbes, Yvonne Perrie

**Affiliations:** Strathclyde Institute of Pharmacy and Biomedical Sciences, University of Strathclyde, Glasgow G4 0RE, UK; maryam.hussain@strath.ac.uk (M.T.H.); neil.forbes@strath.ac.uk (N.F.)

**Keywords:** microfluidics, liposome, solubilisation, protein quantification, reverse-phase-high performance liquid chromatography (RP-HPLC), HPLC-evaporative light scattering detector (HPLC-ELSD)

## Abstract

Advances in manufacturing processes provide the ability for the high throughput production of liposomes containing a range of moieties, from small molecules to large biologicals (including proteins and nucleic acids for prophylactic and therapeutic applications). Whilst rapid quantification methods for small molecules are generally well established, the ability to rapidly quantify liposomal entrapment of proteins is limited. Indeed, most standard protein quantification techniques (including the BCA assay and Reverse phase-high performance liquid chromatography (RP-HPLC)) measure protein encapsulation indirectly, by measuring the amount of non-incorporated drug, and subtracting from the initial amount of protein added. However, this can give inaccurate and misrepresentative results. To address this, we have developed a range of methods to directly quantify protein entrapment within liposomes. The encapsulation efficiency within neutral, anionic and cationic liposome formulations was determined by three techniques; BCA assay, RP-HPLC and HPLC coupled to an evaporative light scattering detector, (HPLC-ELSD). All three methods are reliable for the quantification of protein, with linear responses and correlation coefficients of 0.99, and LOQ for all three methods being less than 10 µg/mL. Here within, we provide three methods for the rapid and robust quantification of protein loading within liposomal (and other bilayer) vesicle systems.

## 1. Introduction

Liposomes are well recognised for their efficacy in drug delivery, with a growing interest within the field of vaccine development [1,2]. The delivery and appropriate targeting of subunit antigens or highly purified protein recombinants as vaccines can be enhanced by incorporating antigens with a suitable delivery system, particularly for routes of administration that pose challenges for the stability of the proteins such as the oral, intranasal or pulmonary [3]. Liposomes are a well-established drug delivery system, and their use as a delivery vehicle for proteins and peptides, in particular for the delivery of vaccines, is widely reported [4,5,6]. However, until recently, the manufacture of liposomal delivery systems has presented a notable barrier in the translation from bench to product [7]. For example, marketed products, such as the Doxil/Caelyx liposomal formulation, requires time consuming, multi-step procedures [8], which incurs high economic costs. However, with an increased interest surrounding novel technologies that use microfluidics to produce liposomal vesicles [7,9,10,11], industrial scale production of liposomes is now much more applicable. Given this rise in high-throughput manufacturing techniques for liposomal delivery vehicles, as well as their growing use as vaccine delivery systems, there remains a need for rapid analytical techniques for the quantification of protein loading within liposomal delivery systems [12]. 

Currently, there are a range of techniques available to quantify the protein loading capacities of drug delivery systems (Table 1) [13,14,15,16,17,18,19]. Protein quantification techniques can include bicinchoninic acid assay (BCA), variations of high-performance liquid-based chromatography (HPLC) and the use of fluorescently labelled or radio-chemically labelled proteins. Less novel chemical analytical techniques also include the use of the Kjeldahl method to determine the nitrogen content in organic substances. The BCA assay uses peptide bonds in the protein reducing Cu^2+^ to Cu^+^ at a rate proportional to the amount of protein present. The bicinchoninic acid reagent then binds with the Cu^+^, forming a complex which absorbs light around 562 nm wavelength, allowing a direct correlation between protein concentration within a sample and absorbance to be made [20]. Whilst the BCA assay can be utilised for large sample screening given the microplate setup, limitations still exist involving interference from a range of agents, including lipids. Other high throughput methods, such as HPLC, can be used to quantify protein, with many variations of HPLC techniques available [21,22], including reverse phase (RP) HPLC, which is commonly used for protein analysis. Although RP-HPLC and the BCA assay are readily available to most analytical laboratories [23], the use of HPLC-evaporative light scattering detector (HPLC-ELSD) is a good alternative for when the active pharmaceutical ingredient does not have a chromophore, or for impurity analysis [24]. Previous papers from our group have shown the ability to quantify lipids using an HPLC-ELSD system [25], and it is reported to detect analytes at high sensitivity rates [26]. 

However, despite these available methods, routinely the encapsulation efficiency within liposomes is determined indirectly by measuring the free un-encapsulated protein following separation via, i.e., centrifugation, dialysis or chromatography. This presents major issues, given it involves the assumption that all of the protein not measured is associated with the delivery vesicles and assumes mass balance is achieved. Therefore, to address this and better support the formulation and development of liposomal protein delivery systems, we have compared the ability of three techniques (BCA, RP-HPLC and HPLC-ELSD) to directly quantify encapsulated protein within liposomal delivery vesicles. 

## 2. Materials and Methods

### 2.1. Materials

The lipids 1,2-distearoyl-sn-glycero-3-phosphocholine (DSPC), 1,2-dioleoyl-3-trimethylammonium-propane (DOTAP) and L-α-phosphatidylserine (Brain PS, Porcine) were all purchased from Avanti Polar Lipids Inc., Alabaster, AL, US. Cholesterol (cholesterol), Ovalbumin (OVA), trifluoroacetic acid and D9777-100ft dialysis tubing cellulose were purchased from Sigma Aldrich Company Ltd., Poole, UK. A Jupiter column (C18 (300 Å), 5 µm, dimensions 4.60 × 150 mm pore size 100 Å) was used in HPLC and purchased from Phenomenex., Macclesfield, UK. The Pierce™ micro BCA Protein Assay kit, HPLC grade methanol and 2-propanol were purchased from Fisher Scientific, Loughborough, England, UK. All water and solvents used were HPLC grade.

### 2.2. Methods

#### 2.2.1. Protein Quantification Techniques

An RP-HPLC method was developed using a universal UV-HPLC by Hewlitt Packard 1100 Series (Santa Clara, CA, USA) to quantify the model antigen ovalbumin (OVA). All samples were run at 280 nm, using a C18 column (i.d. 150 × 4.6 mm) from Phenomenex (Macclesfield, UK). A 1 mL/min flow rate was used with a twenty-minute elution gradient, composed of solvent A (0.1% TFA in water) and solvent B (100% methanol). During the first ten minutes, the gradient was 100:0 (A:B), at 10.1 min 0:100 (A:B) and then back to the initial gradient of 100:0 (A:B) from 15.1 to 20 min. The injection volume for the sample was 20 µL. 

For ELSD, HPLC was used in conjunction with a SEDEX 90LT evaporative light scattering detector (ELSD) (Sedex sedere, Alfortville, France) for OVA quantification. A Jupiter A100 column was used to detect the OVA protein. The flow rate used was 1 mL/min, with a gain of 8 and an OVA peak appearing at 11.8 min. A standard calibration curve for OVA was established using various concentrations; the amount of encapsulated OVA in liposomes was calculated using the peak area of the sample in relation to the standards. 

Protein quantification using Micro BCA (Pierce™ BCA Protein Assay Kit, Sigma Aldrich, Poole, UK) protein assay was carried out under manufacturer’s instructions. Briefly, samples were incubated up to 2 h at 35 °C, with 150 µL of sample + 150 µL of the working reagent. Absorbance was then measured at 562 nm using a Bio-rad 680 microplate reader. 

#### 2.2.2. Liposome Manufacture and Purification

The preparation of liposomes by microfluidics was conducted on the Nanoasemblr^®^ Benchtop system from Precision Nanosystems. Selected lipids were dissolved in methanol at specific concentrations (ranging primarily between 0.1–4 mg/mL total lipid) and injected through one of the two inlets on the microfluidics herringbone micromixer chip, whilst the aqueous phase, PBS; pH 7.3 ± 0.2 or TRIS (pH 7.4) is injected into the second inlet. A flow rate ratio (FRR; the ratio between the aqueous phase and the lipid phase) of 3:1 was selected for neutral and anionic liposomal formulations, while 1:1 FRR was selected for cationic formulations. Total flow rates (TFR) (the speed at which the two inlets are injected through the chip) between 10–15 mL/min were selected. For OVA loaded liposomal samples, OVA is added in the aqueous phase at specific concentrations and the same principles for the production of empty liposomes was followed. 

Following microfluidic production of liposomes, purification of solvent from the sample is required. For “empty” liposomes, dialysis was conducted using *M*_w_ 14,000 Da membrane, where 1 mL of the liposomal sample (0.1–4 mg/mL) was loaded and sealed, before being submerged in 200 mL of equivalent buffer for 1 h at room temperature under gentle agitation via magnetic stirring. The dialysis membrane was pre-treated in a solution of 2% sodium bicarbonate, 1 mM EDTA and 1 L of ultrapure water at 80 °C for 2 h under magnetic stirring. The membrane was then rinsed with water to remove any trace of pre-treated solution and stored in 20% ethanol (EtOH). For purification of OVA loaded liposomes, samples were purified using Krosflo Research Iii tangential flow filtration system fitted with an mPES (modified polyethersulfone) column with a pore size of 750 kDa to support effective separation of the protein (OVA; 45 kDa) and the liposomes. Liposomal samples were circulated through the column and purified through difiltration, with fresh PBS being added at the same rate as the permeate leaving the column.

#### 2.2.3. Method Validation

Linearity was assessed by the design of calibration curves across ovalbumin concentrations. The signal output (Area (mAU), Area (mV) and absorbance for RP-HPLC, ELSD-HPLC and BCA respectively) was plotted against known concentrations to determine the equation of the straight line and regression coefficient (*R*^2^).

Limit of detection (LOD) and limit of quantification (LOQ) were calculated using the following Equations (1) and (2). The standard deviation of the response (σ), divided by the gradient of the slope, multiplied by 3.3 or 10 (LOD and LOQ, respectively) [33,34].

(1) $$LOD=\left(3.3\times \left(\frac{\sigma }{S}\right)\right)$$

(2) $$LOQ=\left(10\times \left(\frac{\sigma }{S}\right)\right)$$

Accuracy (trueness) was calculated using the difference between theoretical and experimental values, taken at three separate concentrations across the assay in triplicate using a low, medium and high concentration value [22,33]. The accuracy can be defined as the closeness of agreement between the mean and the accepted true value together with confidence values [33].

(3) $$Accuracy=\frac{\left(True\;Value-measured\;value\right)\times 100}{True\;Value}$$

Intra-day Precision (Repeatability) is an expression of the closeness of values taken under the same experimental conditions, over a short period of time (the same day). Inter-day Precision (Intermediate Precision) was determined over 3–5 separate days. Results are expressed as %RSD using a minimum of nine determinations (three concentrations: low, medium and high, three replicates of each). 

(4) $$\%\;RSD=\left(\frac{Standard\;deviation}{mean}\right)\times 100$$

#### 2.2.4. Statistical Analysis

Results are represented as mean ± SD with *n* = 3 independent batches. ANOVA tests were used to assess statistical significance (*p* value of less than 0.05), with a Bland and Altman method used to assess the agreeability between the three quantification methods. The mean difference, along with the standard deviation, was calculated with a 95% confidence interval. 

## 3. Results

**Figure 1:**
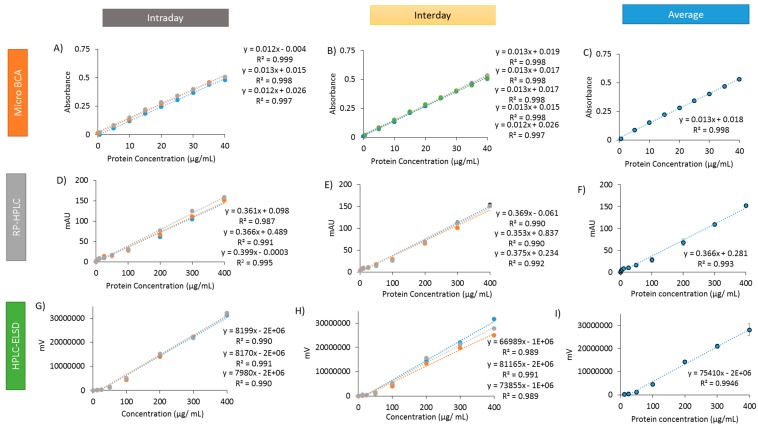
Ovalbumin calibration curves for three quantification techniques. The calibration curves for micro BCA (**A**–**C**) include: Intraday curves (**A**), Interday curves (generated over 5 separate days) (**B**), alongside the average (**C**). Calibration curves were also generated for RP-ELSD (**D**–**F**), including: Intraday curves (**D**), Interday curves (generated over 3 separate days) (**E**), as well as the average (**F**). The same was also generated for the HPLC-ELSD (**G**–**I**): Intraday curves (**G**), Interday curves (**H**), and the average (**I**). Results represent the average of at least three replicate measurements ± standard deviation (SD).

Calibration curves were generated using the micro BCA assay, RP-HPLC and HPLC-ELSD to establish linearity (Figure 1), and the validation results from each process are summarised in Table 2. 

### 3.1. Ovalbumin Quantification via Micro BCA Assay

Calibration curves were generated by micro BCA analysis across a concentration range of 0.5–40 µg/mL (Figure 1A–C). A linear relationship was observed as expected (*R*^2^ values > 0.997), with Figure 1A showing the intra-day repeatability, Figure 1B showing inter-day precision and Figure 1C showing the average. The LOD and LOQ were 1.85 µg/mL and 5.61 µg/mL, respectively (Table 2). Intra-day repeatability and inter-day precision (over five independent days) was calculated at low (5 µg/mL), medium (20 µg/mL) and high (35 µg/mL) concentrations. At each concentration, the mean value and the % RSD was calculated. At each concentration, both intra- and inter-day precision was acceptable, with the %RSD being with ±5% (Table 2). Accuracy was also calculated across the three concentration ranges, with all determinations falling within an accepted range (95–105%; Table 2).

### 3.2. Ovalbumin Calibration Curves Using RP-HPLC

Using RP-HPLC, a gradient method was used to establish a calibration curve (from 5–400 µg/mL) (Figure 1D–F). Again a linear relationship was observed ((*R*^2^ values > 0.987) with good intra-day (Figure 1D) and inter-day (Figure 1E) reproducibility. Using this method, LOD and LOQ values of 2.43 µg/mL and 7.37 µg/mL, respectively, were determined (Table 2). Intra-day repeatability and inter-day precision (over five independent days) was calculated at low (25 µg/mL), medium (200 µg/mL) and high (400 µg/mL) concentrations. At each concentration, the %RSD being with ± 10% (Table 2). Accuracy was also calculated across the three concentration ranges, with all determinations falling within an accepted range (95–105%; Table 2). 

### 3.3. Ovalbumin Calibration Curves Using HPLC-ELSD

The ELSD was used together with the HPLC to determine if this technique was comparable to the RP-HPLC method. As with the RP-HPLC method, a gradient elution method was used. Calibration curves were established (using the concentration ranges 0–400 µg/mL; Figure 1G–I). Intra-day and inter-day repeatability curves were again plotted (Figure 1G,H), with the regression coefficient greater than 0.989 for all curves (Table 2). Using this method, the LOD was 0.77 µg/mL and LOQ was 2.33 µg/mL. Similar to RP-HPLC, the intra-day and inter-day precision was calculated at low, medium and high concentrations (Table 2). Intra-day repeatability RSD values remained within the acceptance criteria (at 5% RSD or below). With inter-day repeatability, the RSD values increased up to 11.44% (Table 2). Accuracy of the assay across the concentrations remained within the acceptance criteria, although at the lower concentration of 100 µg/mL the accuracy dropped to 93.37 ± 4.41%. 

### 3.4. Liposome Solubilisation and Lipid Interactions

**Figure 2:**
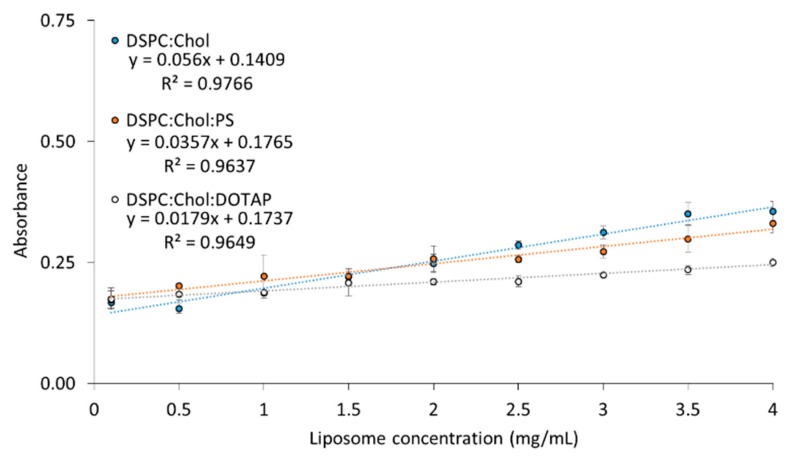
The effect of increasing liposome concentration on micro BCA absorbance with no ovalbumin added. Three liposomal formulations were produced using microfluidics (FRR 3:1 and 1:1, TFR 10 mL/min), DSPC:Chol, DSPC:Chol:PS and DSPC:Chol:DOTAP and assessed for BCA absorbance interference. Results represent the average of at least three replicate measurements ± standard deviation (SD).

Given all three methods were shown to effectively quantify protein concentration, the next step was to develop an extraction method for measuring protein within the liposomes and also to consider interference from lipids within the formulation. In order to quantify the amount of entrapped protein inside liposomes, solubilisation of the liposomes was required. To achieve this, a previously reported and validated protocol using isopropanol (IPA):buffer (at a 50/50 *v*/*v*) was adopted [35]. To investigate the impact of liposome interference, three formulations were selected. A neutral liposomal formulation (DSPC:Chol), an anionic formulation (DSPC:Chol:PS) and the cationic liposomal formulation (DSPC:Chol:DOTAP). The formulations were prepared by microfluidics at a range of final lipid concentrations (0.1–4 mg/mL). In the case of RP-HPLC, the presence of lipids does not interfere with the quantification of the OVA, but solubilisation of the liposomes is necessary, and thus the IPA:buffer 50:50 *v*/*v* was adopted. Due to the nature of the ELSD, the encapsulated OVA can be quantified without solubilisation of the liposomes, and the lipids do not interfere in protein quantification. In the case of the BCA assay, solubilisation of the liposomes is required to release the protein, and the presence of lipids also interferes with the assay. The results in Figure 2 show a gradual increase in absorbance for all three formulations as the concentration increases, with all three formulations showing high degrees of linearity (*R*^2^ > 0.96). A small gradient for all three formulations can be observed, with lipid concentration having limited impact across the range tested (Figure 2). However, the degree of interference varied with formulation, with the cationic liposomal formulation showing the lowest increase over the concentration range tested, whilst the anionic and neutral formulation resulted in higher levels of interference (Figure 2). 

**Figure 3:**
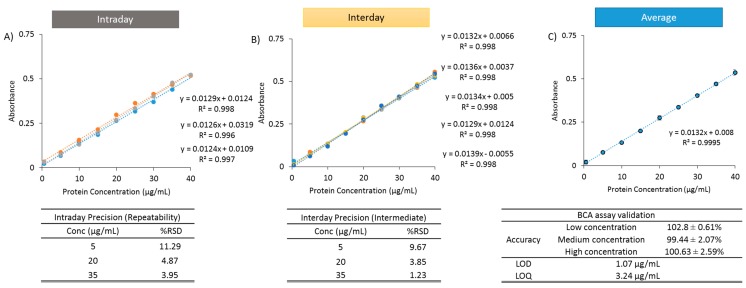
Ovalbumin calibration curves in water in the presence of liposomes (DSPC:Chol 10:5 wt/wt; at a fixed final concentration of 1 mg/mL). The LOD and LOQ values were established using micro BCA, following empty liposome blank removal. Intraday curves were generated (**A**) within the same day, while Interday curves were generated over 5 separate days (**B**) along with the average (**C**). Accuracy was determined at three concentrations, while Intra-day and Inter-day precision was calculated across three different concentrations, with %RSD shown.

Lipid interference in bicinchoninic acid based assays has been previously shown by Kessler and Fanestil [36]. Therefore, in order to determine whether OVA can be quantified in a linear manner in the presence of liposomes, “empty” DSPC:Chol (10:5 wt/wt, 3:1 FRR, 10 mL/min) liposomes were produced via microfluidics and then mixed with the OVA at a final concentration of 1 mg/mL. Protein concentrations remained fixed as for OVA in water (Figure 1), and the resulting blanks for subtraction consisted of liposomes in water with no OVA present. Figure 3 shows the intra-day repeatability (3 replicates; Figure 3A), the inter-day repeatability (5 days; Figure 3B), and the average of three calibration curves used generated for LOD and LOQ is shown in Figure 3C. All curves generated *R*^2^ > 0.99, and despite the presence of liposomes in the samples, the LOD and LOQ values following blank subtraction remained low (1.07 and 3.24 µg/mL, respectively), similar to the micro BCA analysis of protein in water alone (Figure 1). Accuracy values were also within the accepted range (102.8 ± 0.61, 99.44 ± 2.07 and 100.63 ± 2.59% at low, medium and high concentrations, respectively), and intra-day and inter-day repeatability %RSD values of 5% and below were obtained for both medium and high concentrations (Figure 3). Intra-day and inter-day precision resulted in a high %RSD value for the lowest concentration of 5 µg/mL (11.29% and 9.67%, respectively); however, the 20 and 35 µg/mL values remained within the acceptance criteria (Figure 3). These results show that the presence of protein outside of liposomes can be measured using the BCA assay if appropriate liposome blanks are incorporated. This method could be used for the indirect quantification of protein loading, however, as mentioned, this method is limited due to assumptions that no protein is lost during the production process.

**Figure 4:**
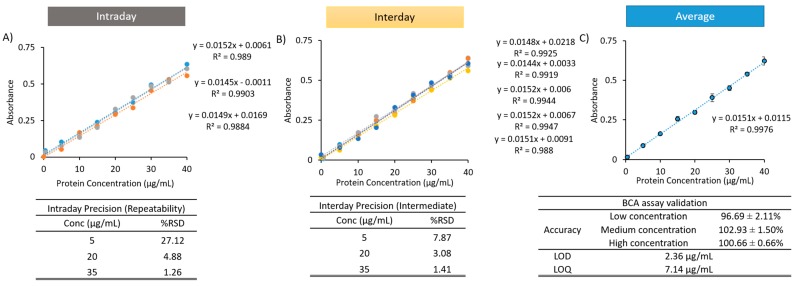
Ovalbumin calibration curves in water in the presence of liposomes (prepared as per Figure 3) and the addition of solubilisation mixture (50/50 *v*/*v* IPA/water). The LOD and LOQ values were established using micro BCA. Intraday curves were generated (**A**) within the same day, while Interday curves were generated over 5 separate days (**B**) along with the average (**C**). Accuracy was determined at three concentrations, while Intra-day and Inter-day precision was calculated across three different concentrations with, %RSD shown.

Given the presence of lipid could be accommodated within the assay, the next stage was to determine the protein entrapment efficiency following solubilisation to allow the direct quantification of protein encapsulated within liposomes. Therefore, calibration curves were established (using the same concentration ranges of 0–40 µg/mL) to determine whether linearity can be achieved in the presence of solubilisation mixture and liposomes using micro BCA. Figure 4 shows the intra-day repeatability (3 replicates; Figure 4A), the inter-day repeatability (5 days; Figure 4B), and the average of three calibration curves used generated for LOD and LOQ is shown in Figure 4C. Again, all curves resulted in regression coefficients greater than 0.98. With the presence of the solubilising agent, the LOD and LOQ (2.36 and 7.14 µg/mL, respectively) was found to be higher than protein alone (Table 2). Intra-day and inter-day repeatability RSD values for both medium and high concentrations remained within the acceptance criteria (5% RSD or below), however, low concentrations of intra-day and inter-day repeatability resulted in a calculated %RSD of 27.12% and 7.87%, respectively (Figure 4). Accuracy of the assay at low, medium and high concentrations all remained within the acceptance criteria. With the micro BCA assay, the manufacturers note that if interfering substances cannot be purified, the sample can be diluted down to an acceptable level of interference. In the liposome formulations tested here (and specifically the concentrations used), linearity could be maintained. However, the addition of up to 5% SDS can also be considered if the lipid concentration is high within the sample and a loss of linearity is observed.

### 3.5. Comparing the Analytical Techniques for the Quantification of OVA Loaded Liposomes

**Figure 5:**
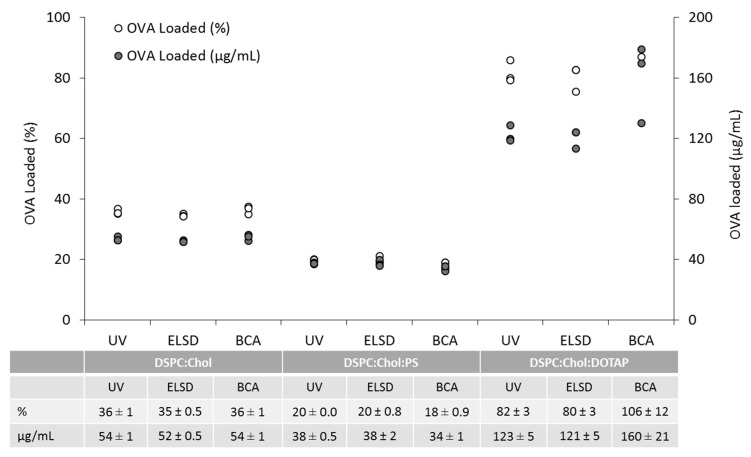
Comparative study between three protein quantification techniques, RP-HPLC, HPLC-ELSD and BCA assay. All three liposomal formulations were made using microfluidics. The DSPC:Chol and DSPC:Chol:PS formulations were made at a 3:1 FRR and 15 mL/min TFR (4 mg/mL initial lipid and 0.25 mg/mL initial ovalbumin concentration). The DSPC:Chol:DOTAP formulation was produced at a 1:1 FRR, and the ovalbumin was adsorbed onto the surface by passing pre-made DSPC:Chol:DOTAP formulation through the microfluidics NanoAssemblr. All results were measured three times, with the average encapsulation and ovalbumin loading calculated.

**Figure 6:**
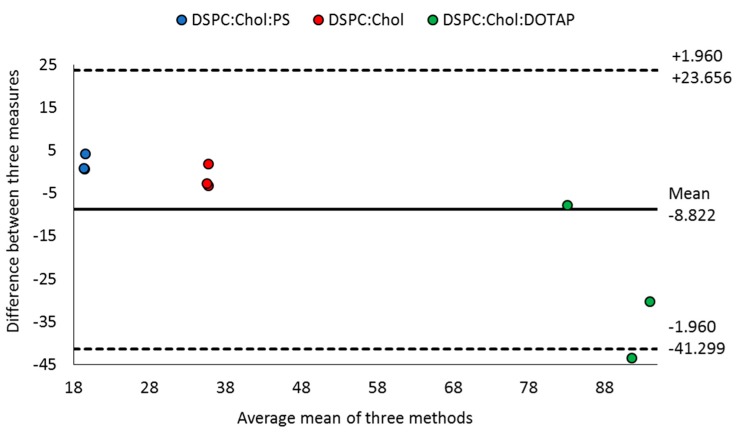
Bland and Atlman plot analysis for the comparison of three analytical techniques. Plot of differences between three analytical techniques (RP-HPLC, HPLC-ELSD and BCA assay) on the y-axis, versus the mean of the three analytical techniques for the three formulations (DSPC:Chol, DSPC:Chol:PS and DSPC:Chol:DOTAP). The calculated mean is −8.8 (horizontal solid line), with the bias represented by the gap between the mean and the dashed lines. All formulations were measured three times for the encapsulation efficiency, with each measurement plotted.

With all three methods established, the ability of the three analytical techniques (BCA assay, RP-HPLC and HPLC-ELSD) to quantify entrapped OVA was analysed. Three liposomal formulations containing DSPC lipid were investigated in triplicate (Figure 5). All three methods are able to quantify the amount of OVA encapsulated by neutral DSPC:Chol liposomes (Figure 5), with the amount calculated being between 52–56 µg/mL (represented by grey circles in Figure 5), with an encapsulation efficiency of 34–38% (open circles). Similar reproducibility was shown with anionic (DSPC:Chol:PS) and cationic (DSPC:Chol:DOTAP) formulations. However, with the cationic liposome formulation, where the protein is electrostatically bound to the liposomal membranes, the micro-BCA assay gave a broader range of encapsulation efficiencies (87–119%; mean 106 ± 12%; Figure 5) compared to the HPLC based methods. In contrast, the efficiency measured using the RP-HPLC was 82 ± 3%, and with HPLC-ELSD it was 80 ± 3%. Statistical analysis of the three techniques showed the results obtained are not significantly different (*p* < 0.05), which confirms the agreeability between the three techniques. Further analysis comparing the techniques was performed using the Bland and Altman plot analysis (Figure 6). This is an alternative analytical approach; it is used to quantify the agreeability between different methods by calculating the mean difference and identifying the limits of agreement [37]. All three formulations were added into the same figure for visual comparison, with the upper and lower limits of agreement calculated as a whole. From Figure 6 there is no obvious bias, with all formulation plots within the upper and lower limits of agreement. The neutral DSPC:Chol and anionic DSPC:Chol:PS formulation plots are close to the mean, highlighting a good degree of agreeability between the three methods. In comparison, the DSPC:Chol:DOTAP plots are more variable with a wider distribution, which highlights the variability between the three cationic formulation batches (as highlighted in Figure 5) rather than the analytical techniques used. As the OVA is adsorbed onto the surface rather than encapsulated within, this may result in less effective solubilisation and release of the protein from the formulation, leading to a greater degree of variability between the formulations produced. Overall, the statistical results show the methods are comparable to one another, with all three analytical techniques producing similar encapsulation results.

## 4. Discussion

Whilst there are a range of analytical tools available to quantify protein concentration, the ability of these methods to quantify protein loading within liposomes has received limited robustness testing. Therefore, a deeper understanding of the advantages and limitations of these assays is necessary for their effective use. During formulation development, small quantities of liposomes containing protein are produced, with assays requiring a high degree of sensitivity for quantification. Initial experimentation assessed the ability of three techniques (RP-HPLC, HPLC-ELSD, BCA assay) to determine ovalbumin concentrations. All techniques showed high degrees of linearity over the protein concentrations tested (*R*^2^ > 0.98) (Figure 1), with accuracy and precision values within ICH guidelines. All three methods have the ability to detect and quantify protein at low concentrations, with LOD values of less than 3 µg/mL and a LOQ values of between 2.33–7.37 µg/mL. The ELSD-HPLC method is the most sensitive, with the lowest LOD (0.77 µg/mL) and LOQ (2.33 µg/mL) calculated. An added feature of the ELSD-HPLC is the ability to change the gain (the sensitivity) of ELSD component. This allows the system to be further optimised, making this system more versatile and easily adaptable depending on specific needs. 

As the liposomes are a mixture of lipids, cholesterol and protein, the quantification of protein without interference from the lipid components or solubilisation is important. Whilst the lipids do not interfere with the RP-HPLC and HPLC-ELSD techniques, interference is observed from lipids while using BCA [36], resulting in higher absorbance values. To address this, we have developed a method to accommodate and circumvent lipid background interference, and in the liposome concentration range tested here, linearity was maintained. However, at much higher liposome concentrations the assay could potentially lose its linear association between absorbance and protein concentration as a result of the lipid interference. When attempting to analyse protein loading, it is crucial that relevant liposome blanks are produced in order to accurately quantify the protein within the sample. However, based on Figure 2, the background interference for a given liposome formulation at a given concentration can be calculated. Therefore, when analysing a large number of samples with the same liposome concentration and formulation, the BCA assay can be used as a valuable tool for high throughput screening.

In comparison, the HPLC techniques offer quick quantification time, in addition to having the capacity to scale-up to larger quantities. Both RP-HPLC and HPLC-ELSD offer ease of quantification, as the lipids do not interfere with the ovalbumin quantification. Previous results comparing HPLC separation modes (including reverse phase and size exclusion chromatography) have found all HPLC separation techniques are good for high precision quantification of free ovalbumin. The reverse phase HPLC method is a robust method, as shown here and in previous studies [38]. The results from our studies show the RP-HPLC (reverse phase method) is a good quantification method for protein, with an LOQ of less than 10 µg/mL determined. Equally, the HPLC-ELSD quantification performed to a high degree of accuracy (>90%) and is the most sensitive method (LOQ of 2.33 µg/mL). Unlike the BCA assay method, solubilisation of the liposomes is not necessary, due to the vaporisation of the analytes in HPLC-ELSD, and that quantification of lipids and proteins may be undertaken simultaneously. Comparison of the three quantification techniques has shown all three methods (RP-HPLC, HPLC-ELSD, BCA assay) are agreeable. The ANOVA results show there is no significant difference between the three techniques when measuring OVA encapsulation. However, there is an inherent error associated with measuring variables (such as encapsulation efficiency); neither gives an absolute correct measurement. Linear regression models are not favoured when comparing methods, as they study the linear relationship between measurements [39]. As a result, the Bland and Altman approach was used to measure the comparability between the three analytical techniques. It is based on the agreement between methods by studying the mean difference and setting limits of agreement [40,41]. The data points are plotted as a scatter plot and are within agreement with 95% of the data being within ± 2 standard deviations [41,42]. This is observed irrespective of the formulation investigated (DSPC:Chol, DSPC:Chol:PS and DSPC:Chol:DOTAP), thus highlighting good agreeability between the three analytical techniques. These results highlight the versatility of all three techniques to quantify protein loading in neutral, anionic and cationic charged liposomes. Whilst pegylated liposomes were not tested within this study, the above outlined methods will also be applicable to such formulations. Furthermore, although all three methods can be used for protein quantification, the technique and separation method will depend on the protein and formulation to be investigated [38,43]. 

## 5. Conclusions

The three analytical techniques (BCA assays, RP-HPLC and HPLC-ELSD) were compared for their ability to determine OVA concentration. The results are comparable, with all techniques able to detect and quantify OVA to a high degree of sensitivity. HPLC techniques are preferred for larger screening as the processing time is quicker. In particular, HPLC-ELSD can be fine-tuned by adjusting the gain, with samples not requiring solubilisation, and thus, may be the most ideal for the quantification of protein loaded in liposomes. Should solubilisation be required, the outlined method was shown to be effective for neutral, anionic and cationic liposomes. Depending on the protein encapsulated, the HPLC methods may need optimising depending on the protein attributes. The final analytical choice, however, will also be dictated by time and the resources available.

## Figures and Tables

**Table 1:** Examples of methods used to quantify protein loading in liposomal formulations.

Protein Loaded	Liposome Formulation	Liposome Production Technique	Method of Protein Quantification	Reference
Bovine Serum Albumin	Dipalymitol phosphatidylcholine, cholesterol and didodecyldimethyl ammonium bromide.	Lipid film hydration	BCA Assay	[13]
Ovalbumin	Phospholipid S and cholesterol.	Lipid film hydration	BCA Assay	[15]
Hepatitis B core peptide (HBCAg126-140)	Dipalymitol phosphatidylcholine, cholesterol and dipalmitoyl phosphatidylglycerol.	Modified Freeze-Thaw method	RP-HPLC	[16]
Superoxide Dismutase	A range of cationic liposome systems were tested.	Lipid film hydration	HPLC	[17]
Bovine Serum Albumin	Phosphatidylcholine and cholesterol based formulation.	Lipid film hydration	Kjedahl Method	[27]
Acetylcholinesterase	Egg phosphatidylcholine based formulation.	Lipid film hydration	Acetylcholinesterase activity	[28]
Insulin	Hydrogenated Phosphatidylcholine and cholesterol.	Lipid film hydration	BCA Assay	[29]
Protein corona	PEGylated Doxorubicin-encapsulated Liposomes	Lipid film hydration	BCA Assay	[30]
Epidermal Growth Factor (EGF)	Dimethyldioctadecylammonium bromide	Lipid film hydration	HPLC	[31]
Emtansine (antibody–drug conjugate)	Distearoylphosphatidylethanolamine-poly, 1-2-dioleoyl-sn-glycero-3-phoshoeethanolamine	Lipid film hydration	HPLC	[32]

BCA: bicinchoninic acid assay; RP-HPLC: reverse –phase high performance liquid chromatography; HPLC: high performance liquid chromatography.

**Table 2:** A comparison of three protein quantification techniques; micro BCA, RP-HPLC, and HPLC-ELSD. For all three methods, the accuracy was determined at three concentrations, while Intra-day and Inter-day precision was calculated across three different concentrations with %RSD shown. The LOD and LOQ were also determined for the respective quantification techniques. Results represent the average of at least three replicate measurements ± standard deviation (SD).

Concentration	*R* ^2^	Equation	Mean (SD)		%RSD		Accuracy	LOD	LOQ
(µg/mL)			Intra-Day	Inter-Day	Intra-Day	Inter-Day	(%)	(µg/mL)	(µg/mL)
micro BCA									
5	0.9985	*y* = 0.013*x* + 0.0178	4.89 (0.75)	4.48 (0.73)	4.40	2.26	95.32 ± 0.94	1.85	5.61
20			20.86 (1.84)	20.61 (1.46)	2.42	1.18	102.94 ± 1.85		
35			35.5 (0.79)	35.1 (0.79)	1.76	0.38	100.63 ± 0.99		
RP-HPLC									
25	0.9925	*y* = 0.3658*x* + 0.2811	26.02 (1.82)	27.87 (1.26)	6.41	4.35	101.39 ± 0.10	2.43	7.37
200			193.15 (18.22)	185.25 (4.24)	9.32	2.28	94.53 ± 6.03		
400			436.44 (9.08)	415.40 (5.00)	2.07	1.20	102.74 ± 3.45		
HPLC-ELSD									
100	0.9946	*y* = 75,410*x* −2 × 10^6^	81.17 (3.33)	87.18 (6.93)	5.89	11.44	93.37 ± 4.41	0.77	2.33
200			203.32 (5.53)	215.08 (7.90)	3.09	4.19	102.31 ± 13.8		
400			417.02 (5.57)	402.31 (36.95)	1.42	9.84	99.21 ± 5.59		
